# Opportunities, risks and policy implications of using health facility mortality data to inform DRG payment design under Ghana’s NHIS

**DOI:** 10.1038/s44401-026-00162-4

**Published:** 2026-11-04

**Authors:** Regina Titi-Ofei, Doris Osei Afriyie, Hillary Kipruto, Justice Nonvignon, Clementine Rossier, Anthony Adofo-Ofosu

**Affiliations:** 1 Institute of Global Health, Faculty of Medicine, Université de Genève (UNIGE), Geneva, Switzerland; 2 Health Finance Department, The Global Fund to fight AIDS Tuberculosis and Malaria, 1218 Geneva, Switzerland; 3 Department of Africana Studies and Global Public Health, Binghamton University, State University of New York, Binghamton, NY USA; 4 Health Information Systems Unit, World Health Organization Regional Office for Africa, Brazzaville, Congo; 5 University of Ghana, School of Public Health, Department of Health Economics, Accra, Ghana; 6 Management Sciences for Health, Arlington, VA, USA; 7 Institut de Démographie et Socioéconomie, Université de Genève (UNIGE), Geneva, Switzerland; 8 Department of Health Policy, University of Health and Allied Sciences, Ho, Ghana; 9 Department of Community Health, School of Medicine, University of Health and Allied Sciences, Ho, Ghana

**Keywords:** Diseases, Health care

## Abstract

Using mortality data to inform Diagnosis-Related Group (DRG) design offers an opportunity to make provider payment systems more responsive to differences in clinical severity and resource needs. Mortality data could support refinement of case-mix classification and cost calibration, while providing a complementary signal for quality monitoring. Using Ghana as an example, this paper explores how facility-level mortality data could enhance the country’s maturing G-DRG system and inform broader reforms across low- and middle-income settings.

## Introduction

Provider payment reforms remain central to the pursuit of universal health coverage (UHC), particularly in low- and middle-income countries (LMICs) that face the dual challenge of rising health needs and constrained fiscal space^1^. Payment mechanisms shape provider behavior by influencing the volume, type, and quality of services delivered, as well as the overall efficiency and sustainability of financing systems^2^.

Over the past two decades, countries have moved away from passive, input-based reimbursement models toward strategic purchasing approaches that link financing to service outputs, efficiency, and quality. In high-income settings, payment innovations have been used to align provider incentives with value rather than volume^3^. Low- and middle-income countries (LMICs) are increasingly following this trajectory- adapting global lessons to local contexts marked by limited fiscal space, fragmented financing, and increasing  epidemiological complexity^1^. The World Health Organization and the World Bank have both emphasized that reforms to provider payment systems are critical for improving allocative and technical efficiency, ensuring accountability for results, and containing costs without compromising access or equity^4^.

In high-income countries, DRG systems typically incorporate explicit measures of severity of illness and risk of mortality through secondary diagnoses, comorbidity indices, and patient-level cost accounting. Systems such as All Patient Refined DRGs (APR-DRGs) or severity-adjusted DRG variants rely on detailed clinical documentation, standardized ICD coding practices, and validated comorbidity measures, with tariffs calibrated using routinely updated hospital cost accounting systems^5^. By contrast, Ghana’s G-DRG system operates in a context of limited diagnostic granularity, incomplete coding of comorbidities, insufficient linkage between clinical outcomes and payment design, and constrained availability of patient-level cost data. As documented in other low- and middle-income settings, DRG systems under these conditions tend to rely on simplified case groupings and historically negotiated tariffs, limiting their ability to adequately capture clinical severity and outcome risk^6,7^.

In the context of such approaches to DRG calibration and nascent facility-level mortality data systems, mortality data can serve as a diagnostic signal to identify poorly specified DRG groupings, disaggregate severity gradients, and identify systematic mismatches between reimbursement levels and clinical risk. Importantly, we do not propose that observed mortality should directly determine reimbursement; rather, mortality can be used to identify DRGs in which existing classifications inadequately capture differences in clinical severity and expected resource use. Similar uses of outcome indicators as signals for payment system refinement have been documented in early DRG reforms in middle-income countries^8,9^.This distinction is therefore critical for Ghana and for other countries seeking to strengthen strategic purchasing while their health information and costing systems continue to mature. This paper explores the opportunity for leveraging the principles of DRG cost calibration utilized in high income contexts but adapting these to low-income contexts that may not have all the requisite data frameworks for such precise costing using outcomes data.Applying these improvements in the development of provider payment mechanisms reflects a broader shift toward value-based purchasing, in which routine health data such as mortality, morbidity, and utilization statistics can be used to optimize tariffs, refine case mix classifications, and strengthen accountability for outcomes^6^.

The availability of improved health information systems and facility-level reporting therefore has expanded opportunities to refine payments not only on inputs and activities but also on the quality and results of care delivered. In Ghana, the Diagnosis-Related Group (G-DRG) payment system was introduced in 2008 and represented a landmark effort to move away from open-ended fee-for-service reimbursement model under the National Health Insurance Scheme (NHIS) and towards a more structured approach that could enhance transparency, predictability, and cost containment^10^. The G-DRG system covers inpatient and bundled outpatient services, while pharmaceuticals remain reimbursed separately under a fee-for-service approach^11,12^. The NHIS tariff manual, which is periodically updated, sets out reimbursement rates for over 550 clinical groupings, ranging from obstetric care to chronic disease management^13^.

Designing a robust DRG system requires attention to several interlinked factors—including accurate clinical classification, reliable cost and utilization data, and appropriate adjustment for case severity and comorbidities. In the absence of such refinements, DRGs may mask meaningful differences in patient complexity and inadvertently create incentives for under-provision of health services or upcoding^14^. Patterns of in-facility deaths and case fatality rates across DRG categories can help identify heterogeneous or poorly specified groups, flag quality differentials, and identify areas where existing classifications may inadequately capture clinical risk^15^. Using mortality data to inform DRG refinement could therefore support more accurate case-mix classification while also strengthening accountability for outcomes and shifting to a stronger value-based purchasing model^7^. Ghana’s G-DRG system has, however, faced challenges and tariffs have sometimes failed to reflect the true cost of treating severe or complex conditions, with criticisms for  insufficient alignment with disease burden or quality outcomes^6^.

At the same time, Ghana’s health information system is evolving. The introduction of electronic claims (e-claims), improvements in hospital reporting, and mortality audits have expanded the availability of facility-based data, including cause-specific mortality. These advances are underpinned by the Ghana Health Information System Strategic Plan (GHISSP)^16^, which seeks to build an integrated, interoperable, and standards-based health information ecosystem. The GHISSP emphasizes strengthening data governance, interoperability, and linkages across routine health information systems, civil registration, and insurance databases to enhance the generation and use of quality data to drive smarter decision-making. While these datasets remain incomplete and uneven in quality, they present a largely untapped opportunity: using facility-level mortality information to refine DRG groupings and strengthen accountability for outcomes. Mortality-informed DRG configuration could improve the homogeneity of case groupings, better align financing with disease burdens, and support  improvements in data quality. Yet the approach is not without risks. Weaknesses in mortality reporting, representativeness biases, and risks of gaming or manipulation could undermine the integrity of the payment system if not carefully managed^17,18^.

In this paper, we examine the potential of utilizing facility-based mortality data to optimize Ghana’s G-DRG payment system. We argue that while this approach holds significant promise for enhancing NHIS sustainability and accountability, its success depends on phased implementation, robust data governance, and deliberate efforts to mitigate equity risks.

### The current landscape of Ghana’s G-DRG system

The G-DRG was introduced to address widespread dissatisfaction with the inefficiencies of fee-for-service reimbursement^19^. Under fee-for-service, providers had strong incentives to over-provide services, leading to escalating costs for the NHIS. The G-DRG was expected to reduce perverse incentives, promote cost control, and enable more predictable expenditure management^10^.

Under the National Health Insurance Scheme, the G-DRG payment mechanism applies to all NHIS-accredited public, private-for-profit, and faith-based providers delivering covered services^20,21^. Primary health centres, district hospitals, regional hospitals, and teaching hospitals are all reimbursed under the G-DRG system, although the complexity of cases treated varies substantially across levels of care^12^.

Tariffs are determined through periodic revisions led by the National Health Insurance Authority (NHIA), drawing on historical cost studies, claims utilization patterns, and stakeholder negotiations^13^. These revisions are constrained by fiscal pressures, limited availability of facility-level cost data, and broader macro-economic considerations. This is further compounded by weaknesses in clinical and intervention coding: incomplete and inconsistent use of ICD standards for diagnoses and causes of death, alongside limited adoption of the International Classification of Health Interventions (ICHI), reduces the system’s ability to distinguish case severity, resource intensity, and outcomes across DRG categories^23^. As a result, tariff calibration and claims verification rely on rough groupings that mask variation in clinical complexity and mortality risk, constraining effective DRG refinement^7,24^.

In principle, the DRG system was adopted in Ghana to reward efficiency: health facilities can deliver care at lower cost than the tariff and retain savings, while those that overspend bear the financial risk. However, several structural challenges have undermined the effectiveness of the G-DRG First, G-DRG tariffs have not consistently aligned with prevailing hospital charges and resource requirements, creating potential financing gaps for some services and case types^25^. This is particularly problematic for conditions such as obstetric emergencies, trauma, and advanced HIV/AIDS or TB co-infections, where resource use far exceeds the reimbursement rate^19,22^. Second, claims inflation has persisted, with providers accused of “upcoding” cases to higher-paying DRGs or exaggerating case severity^22^. Third, the G-DRG has been criticized for limited responsiveness to Ghana’s shifting disease burden, including the rise of non-communicable diseases alongside persistent infectious disease burdens^6^.

These limitations underscore the need for refinement. Facility mortality data could provide a new dimension to DRG configuration, offering insights into severity, outcomes, and resource needs that are currently absent from the country’s tariff-setting.

### Opportunities for mortality-informed DRG refinement

The use of mortality data in optimizing the G-DRG system offers several potential benefits for Ghana’s NHIS.

#### Improving clinical homogeneity

A central objective of DRG systems is to group patients with similar clinical characteristics and expected resource use into homogeneous categories. In practice, however, DRG systems in many low- and middle-income countries aggregate clinically heterogeneous cases due to limited diagnostic detail, weak coding of comorbidities, and constrained cost data^6,26^. This heterogeneity undermines both cost accuracy and the ability of payment systems to reflect clinical risk.

Facility-level mortality data can serve as an informative signal of unobserved severity within DRG categories^15,27^. Persistently higher case fatality rates within specific DRGs may indicate that groupings mask substantial differences in disease severity, complications, or treatment intensity. In high-income settings, similar insights are generated through formal severity adjustment mechanisms embedded within DRG systems, which stratify cases by risk of mortality and complications^28^.

 In Ghana, mortality patterns could be used to identify DRGs requiring further clinical stratification, with observable patient characteristics subsequently used to define severity subclasses and associated payment weights. Consistent with established approaches to DRG design and tariff setting, such analyses would be conducted across sufficiently large patient cohorts and facilities and used to inform periodic refinement of DRG classifications, rather than facility-specific payment adjustments. Comparable approaches have been used in early DRG reforms in middle-income countries, where outcome indicators informed iterative revisions to case groupings rather than direct tariff adjustments^8,9^.

#### Aligning  payments with clinical severity and resource needs

Mortality-informed DRG refinements can also help identify where existing payments inadequately reflect differences in clinical severity and resource needs. In Ghana, conditions associated with high mortality such as severe malaria, neonatal sepsis, advanced cancers, and complicated obstetric cases^17^ are often under-reimbursed relative to their resource requirements^19^.

International experience shows that DRG systems that fail to account for severity and outcome risk tend to underpay hospitals treating sicker patients, leading to risk selection and inequitable resource allocation^14^. In several advanced economies, routine hospital data have been used to identify differences in severity and expected resource needs that inform more refined DRG classifications and payment weights^14^.

While Ghana’s data environment does not yet support formal severity weighting, triangulating mortality patterns with utilization and claims data can help prioritize DRGs for revision and ensure that tariffs better reflect the epidemiological transition toward more complex and chronic conditions.

#### Strengthening accountability for quality

Beyond cost calibration, mortality patterns within DRGs can serve as an accountability and quality monitoring tool. Unexpectedly high mortality rates within specific DRGs may signal gaps in clinical quality, referral pathways, or resource availability rather than differences in case mix alone^29,30^.

In high-income health systems, DRG-linked mortality indicators are routinely used for hospital performance benchmarking and quality improvement, even when not directly tied to payment^24^. Similar approaches have been piloted in middle-income settings, where outcome indicators complement claims audits to identify facilities or service lines requiring closer review^8^.

 For Ghana, mortality can serve two distinct functions: informing DRG refinement by identifying inadequately specified case groups, and, when risk-adjusted, serving as an outcome indicator to identify potential quality concerns and support targeted quality-improvement efforts. Higher observed mortality should not result in higher reimbursement, and any introduction of risk-adjusted DRG payments should consequently be implemented alongside performance indicators that monitor quality of care and patient outcomes. This would help ensure that improved adjustment for clinical risk does not inadvertently weaken incentives for quality care, while supporting continuous quality-improvement measures.

### Provider incentives and implementation risks

While facility-level mortality data offers potential value for refining DRG case-mix and improving severity calibration, their use in provider payment design introduces risks related to data quality, representativeness, and provider incentives. In settings where mortality data systems remain incomplete and uneven, these risks may distort reimbursement signals, exacerbate gaming, and undermine equity unless explicitly addressed through careful design and governance.

Recognizing these risks, mortality-informed DRG refinement in Ghana should prioritise phased implementation. We share a few anticipated challenges and risks with implementation.

#### Data quality risks to tariff calibration

Health facility mortality statistics in Ghana remain unreliable^31^. Many facilities lack adequately trained personnel to code causes of death, and misclassification is common due to poor clinical documentation and inconsistent application of ICD standards. Empirical evidence shows that facility death data in Ghana underreport certain causes while overreporting others, reflecting systemic weaknesses in cause-of-death certification and reporting. Without substantial improvements in data quality and completeness, incorporating mortality signals into DRG refinement risks introducing significant distortions into tariff calibration and provider reimbursement.

#### Representativeness bias

Facility-based deaths represent only a subset of overall mortality and are systematically skewed toward more severe cases and populations with access to health facilities^32^. In Ghana, rural populations and poorer households experience a substantial share of mortality outside formal health facilities, particularly for conditions sensitive to primary care access such as childhood malaria and maternal complications. Over-reliance on facility mortality data therefore risks over-weighting tertiary conditions while underrepresenting community-level disease burden. While modelling approaches—such as redistribution of ill-defined causes and calibration against community mortality data—can partially mitigate these biases, failure to account for representativeness limitations could misalign DRG refinements with true population health needs.

#### Perverse incentives and claims inflation

The use of mortality signals in DRG refinement also raises concerns about perverse incentives and strategic provider behaviour. If higher reimbursement is implicitly associated with higher observed mortality or severity, providers may have incentives to misreport diagnoses, exaggerate case severity, or selectively classify patients into higher-paying DRG categories. Directly linking reimbursement to observed deaths should therefore be avoided, as this could also create inappropriate incentives around clinical outcomes. Ghana’s NHIS has already experienced significant claims inflation under the existing G-DRG system, suggesting that mortality-informed DRG refinement, if translated into poorly designed payment adjustments, could intensify these behaviours unless accompanied by robust audit, verification, and accountability mechanisms^22,26^.

#### Institutional capacity constraints

Implementing mortality-informed DRG refinement requires advanced actuarial modelling, epidemiological expertise, and robust IT infrastructure. At present, capacity within the NHIA to undertake such analyses remains constrained, though there is scope to strengthen these functions and draw on lessons from past reforms, including the Ashanti Region capitation pilot. Without adequate technical preparation, institutional coordination, and political buy-in, mortality-informed DRG refinements risk replicating earlier implementation challenges rather than delivering meaningful improvements.

#### Equity risks from differential institutional capacity

Finally, equity risks arise from differential institutional capacity across facilities. Hospitals with stronger digital infrastructure and coding capacity, often located in urban areas, may be better positioned to document severity accurately and benefit from mortality-linked DRG refinements. In contrast, rural and under-resourced facilities may be disadvantaged not because of poorer clinical performance, but due to weaker data systems and limited coding capacity. If unaddressed, these dynamics could exacerbate existing inequities in NHIS financing and access to care, disproportionately affecting facilities serving vulnerable populations^33^.

### Looking forward: a phased and cautious approach

Ghana’s G-DRG system has been a major step in the country’s health financing reform journey, but persistent challenges- including tariff misalignment, rising costs, and weak linkage to outcomes have constrained its effectiveness. Facility-based mortality data represent a promising but underutilized resource that could help refine DRG groupings, better align tariffs with disease burden, and strengthen accountability for quality of care. However, international experience shows that integrating outcome or severity signals into DRG systems requires careful, incremental implementation to avoid unintended consequences such as gaming, cost escalation, or provider resistance.

To maximize benefits while minimizing risks, Ghana should therefore pursue a phased, evidence-based approach to mortality-informed DRG reform. First, mortality-informed DRG refinement should be piloted in a limited set of high-priority clinical areas  such as maternal, neonatal, and selected infectious disease conditions, where mortality data are relatively more complete, and policy relevance is high. Similar piloting approaches that were used in the country when the NHIS explored the implementation of capitation as a provider payment mechanism for the NHIS^34^, and in Thailand and Australia, where severity and outcome adjustments were initially introduced in selected DRG groups before broader system-wide adoption^35,36^.

Second, mortality signals derived from routine facility data should be systematically triangulated with external sources, including Health and Demographic Surveillance System (HDSS) sites (Navrongo, Kintampo, Dodowa), civil registration and vital statistics (CRVS), and modeled estimates from WHO and the Global Burden of Disease. Such triangulation reflects international best practice in contexts where facility deaths are not fully representative of population mortality^32^.

Third, mortality-informed payment reform must be linked to investments in data system strengthening, including ICD-11 training, improved clinical documentation, cause-of-death reporting, and routine mortality audits. Experience from countries with advanced DRG-based systems suggests that payment reforms are most effective when paired with sustained investments in coding quality, audit capacity, and clinical governance^24^. In Ghana, such linkages present an opportunity by which improved data quality can support better tariff calibration, while financing incentives reinforce investments in health information systems.

Fourth, robust safeguards against gaming and unintended provider behavior are essential. Independent coding audits, transparent risk-adjustment models, and alignment with the NHIS e-claims platform can strengthen verification and oversight. Evidence from DRG reforms in both high- and middle-income countries underscores that outcome-linked payments, if poorly designed, may increase incentives for upcoding or selective admission unless accompanied by strong audit and governance mechanisms^8,37^.

Finally, sustained stakeholder engagement is critical for political feasibility and system legitimacy. The NHIA, Ministry of Health, Ghana Health Service, healthcare providers, clinicians and professional associations, policy and health financing experts, academia, and patient and civil society representatives should be involved in the design, piloting, and evaluation of mortality-informed DRG refinement. Ghana’s experience with earlier payment reforms, including the capitation pilot, highlights how limited consultation and trust deficits can undermine technically sound reforms^21,22^.

For Ghana, the next phase of improvements should include continuous learning in order to adopt and adapt carefully, ensuring that mortality data are used as a strategic tool for system improvement. If implemented carefully, mortality-informed DRG refinements could strengthen NHIS sustainability, improve provider payment design, tariff calibration and case-mix adjustment, and better align reimbursement with clinical severity and resource needs, while also catalyzing broader improvements in health information systems, clinical documentation, and mortality surveillance. In this respect, Ghana’s experience demonstrates a practical pathway for other countries in sub-Saharan Africa and similar low- and middle-income settings seeking to align provider payment mechanisms with the realities of disease burden and health system performance.

## Data Availability

No datasets were generated or analysed during the current study.

## References

[1] Gatome-Munyua, A., Sieleunou, I., Sory, O. & Cashin, C. Why is strategic purchasing critical for universal health coverage in sub-Saharan Africa? *Health Syst. Reform* **8**, e2051795 (2022). 10.1080/23288604.2022.2051795 35446198

[2] Kazungu, J. S., Barasa, E. W., Obadha, M. & Chuma, J. What characteristics of provider payment mechanisms influence health care providers’ behaviour? A literature review. *Int. J. Health Plan. Manag.* **33**, e892–e905 (2018). 10.1002/hpm.2565 PMC7611391 29984422

[3] Koricho, M. et al. An Assessment of Provider Payment Mechanisms (PPMs) in ethiopia: implications for redesign of ppms and progress toward universal health coverage. *Health Syst. Reform* **10**, 2377620 (2024). 10.1080/23288604.2024.2377620 39028638

[4] World Bank. *Designing and Implementing Provider Payment Systems- How to Manuals* (World Bank, 2009).

[5] Milstein, R. & Schreyögg, J. The end of an era? Activity-based funding based on diagnosis-related groups: a review of payment reforms in the inpatient sector in 10 high-income countries. *Health Policy* **141**, 104990 (2024). 10.1016/j.healthpol.2023.104990 38244342

[6] Mathauer, I. & Wittenbecher, F. DRG-based payment systems in low- and middle-income countries: implementation experiences and challenges. World Health Organization https://iris.who.int/handle/10665/75150 (2025). 10.2471/BLT.12.115931 PMC3791650 24115798

[7] Mathauer, I., Dale, E., Jowett, M. & Kutzin, J. Purchasing health services for universal health coverage: how to make it more strategic. WHO Health Financing Policy Brief No.6. - References - Scientific Research Publishing https://www.scirp.org/reference/referencespapers?referenceid=3200638 (2019).

[8] Quentin, W., Scheller-Kreinsen, D., Blümel, M., Geissler, A. & Busse, R. Hospital payment based on diagnosis-related groups differs in Europe and holds lessons for the United States. *Health Aff. Proj. Hope* **32**, 713–723 (2013). 10.1377/hlthaff.2012.0876 23569051

[9] Paying for performance in health care: implications for health system performance and accountability. OBS. https://eurohealthobservatory.who.int/publications/m/paying-for-performance-in-health-care-implications-for-health-system-performance-and-accountability (2026).

[10] Schieber, G., Cashin, C., Saleh, K. & Lavado, R. *Health Financing in Ghana* (The World Bank, 2012).

[11] Amporfu, E. & Arthur, E. *Ghana’s Experience with Changing Provider Payment to Capitation in Primary Health Care* (London School of Hygiene and Tropical Medicine, 2022).

[12] Andoh-Adjei, F. X., Nsiah-Boateng, E., Asante, F. A., van der Velden, K. & Spaan, E. Provider preference for payment method under a national health insurance scheme: a survey of health insurance-credentialed health care providers in Ghana. *PLoS ONE* **14**, e0221195 (2019). 10.1371/journal.pone.0221195 PMC6709917 31449530

[13] National Health Insurance Authority. *Ghana National Health Insurance Scheme Tariffs Operational Manual* (National Health Insurance Authority, 2022).

[14] Ellis, R. P. & McGuire, T. G. Provider behavior under prospective reimbursement. *Cost. Shar. Supply J. Health Econ.* **5**, 129–151 (1986). 10.1016/0167-6296(86)90002-0 10287223

[15] Iezzoni, L. *Risk Adjustment for Measuring Health Care Outcomes* (Health Administration Press, 2012).

[16] Ministry of Health. Ghana Health Information System Strategic Plan. Ministry of Health- Ghana https://www.moh.gov.gh/wp-content/uploads/2024/03/HISSP_2022-2025_FINALVERSION-1.pdf (2022).

[17] Byass, P. Cause-specific mortality findings from the Global Burden of Disease project and the INDEPTH Network. *Lancet Glob. Health* **4**, e785–e786 (2016). 10.1016/S2214-109X(16)30203-0 27765285

[18] Setel, P. W. et al. A scandal of invisibility: making everyone count by counting everyone. *Lancet* **370**, 1569–1577 (2007). 10.1016/S0140-6736(07)61307-5 17992727

[19] Agyepong, I. A. & Adjei, S. Public social policy development and implementation: a case study of the Ghana National Health Insurance scheme. *Health Policy Plan.* **23**, 150–160 (2008). 10.1093/heapol/czn002 18245803

[20] Ministry of Health- Ghana. *National Health Insurance Policy Framework for Ghana* (Ministry of Health- Ghana, 2004).

[21] Alhassan, R. K., Nketiah-Amponsah, E. & Arhinful, D. K. A review of the national health insurance scheme in Ghana: what are the sustainability threats and prospects? *PLoS ONE* **11**, e0165151 (2016). 10.1371/journal.pone.0165151 PMC5104458 27832082

[22] Agyepong, I. A. et al. Advancing the application of systems thinking in health: provider payment and service supply behaviour and incentives in the Ghana National Health Insurance Scheme – a systems approach. *Health Res. Policy Syst.* **12**, 35 (2014). 10.1186/1478-4505-12-35 PMC4142471 25096303

[23] Ooi, E. C. W. et al. Facilitators and challenges to ICD-11 implementation: a qualitative study using the consolidated framework for implementation science. *BMC Med. Inf. Decis. Mak.* **25**, 314 (2025). 10.1186/s12911-025-03157-7 PMC12372348 40842009

[24] Busse, R. et al. Diagnosis related groups in Europe: moving towards transparency, efficiency, and quality in hospitals? *BMJ* **346**, f3197 (2013). 10.1136/bmj.f3197 23747967

[25] Peter Gyasi, Darkwa Genevieve Cecilia, Aryeetey Eric, Nsiah-Boateng Ghana’s Diagnosis Related Grouping (G-DRG) tariffs versus hospital charges: implications for inpatient care financing at a secondary hospital in Ghana Cost Effectiveness and Resource Allocation **24** (2026). 10.1186/s12962-026-00747-8 PMC13374358 42174660

[26] Amporfu, E., Agyei-Baffour, P., Edusei, A., Novignon, J. & Arthur, E. Strategic health purchasing progress mapping: a spotlight on Ghana’s national health insurance scheme. *Health Syst. Reform* **8**, e2058337 (2022). 10.1080/23288604.2022.2058337 35695801

[27] Horn, S. D., Sharkey, P. D., Chambers, A. F. & Horn, R. A. Severity of illness within DRGs: impact on prospective payment. *American Journal of Public Health* **75**, 1195–1199 (1985). 10.2105/ajph.75.10.1195 PMC1646367 3929632

[28] Charlson, M. E., Pompei, P., Ales, K. L. & MacKenzie, C. R. A new method of classifying prognostic comorbidity in longitudinal studies: development and validation. *J. Chronic Dis.* **40**, 373–383 (1987). 10.1016/0021-9681(87)90171-8 3558716

[29] Kruk, M. E. et al. High-quality health systems in the Sustainable Development Goals era: time for a revolution. *Lancet Glob. Health* **6**, e1196–e1252 (2018). 10.1016/S2214-109X(18)30386-3 PMC7734391 30196093

[30] Shahian, D. M., Iezzoni, L. I., Meyer, G. S., Kirle, L. & Normand, S. L. Hospital-wide mortality as a quality metric. *Am. J. Med. Qual.* **27**, 112–123 (2012). 10.1177/1062860611412358 21918014

[31] Owusu, A. Y. et al. Institutional mortality rate and cause of death at health facilities in Ghana between 2014 and 2018. *PLoS ONE* **16**, e0256515 (2021). 10.1371/journal.pone.0256515 PMC8425528 34496000

[32] Titi-Ofei, R., Kipruto, H., Atweam, D., Ofosu, A. A. & Rossier, C. Exploring the various sources of mortality estimation in Ghana: a scoping review of data sources, challenges, and opportunities. *Healthcare* **13**, 2331 (2025). 10.3390/healthcare13182331 PMC12469968 41008461

[33] Karamagi, H. C. et al. Towards universal health coverage in the WHO African Region: assessing health system functionality, incorporating lessons from COVID-19. *BMJ Glob. Health* **6**, e004618 (2021). 10.1136/bmjgh-2020-004618 PMC8015798 33789869

[34] Yambah, J. K. et al. The effect of the capitation policy withdrawal on maternal health service provision in Ashanti Region, Ghana: an interrupted time series analysis. *Glob. Health Res. Policy* **7**, 38 (2022). 10.1186/s41256-022-00271-1 PMC9585820 36266718

[35] Tangcharoensathien, V. et al. Achieving universal health coverage goals in Thailand: the vital role of strategic purchasing. *Health Policy Plan.* **30**, 1152–1161 (2015). 10.1093/heapol/czu120 PMC4597041 25378527

[36] Duckett, S. J. Casemix funding for acute hospital inpatient services in Australia. *Med. J. Aust.* **169**, S17–S21 (1998). 10.5694/j.1326-5377.1998.tb123470.x 9830405

[37] O’Reilly, J. & Ward, P. DRG-based Hospital Payment and Efficiency: theory, evidence, and challenges. Open University Press and WHO Regional Office for Europe; 2011 https://esri.ie/publications/drg-based-hospital-payment-and-efficiency-theory-evidence-and-challenges (2026).

